# Substantial batch effects in TCGA exome sequences undermine pan-cancer analysis of germline variants

**DOI:** 10.1186/s12885-019-5994-5

**Published:** 2019-08-07

**Authors:** Roni Rasnic, Nadav Brandes, Or Zuk, Michal Linial

**Affiliations:** 1 The Rachel and Selim Benin School of Computer Science and Engineering, The Hebrew University of Jerusalem, Jerusalem, Israel; 2 Department of Statistics, The Hebrew University of Jerusalem, Jerusalem, Israel; 3 Department of Biological Chemistry, Institute of Life Sciences, The Hebrew University of Jerusalem, Jerusalem, Israel

**Keywords:** Cancer predisposition, TCGA, Germline variants, Batch effect, Somatic mutations, Personalized medicine, Next generation sequencing, BRCA1, Genomic sequencing centers

## Abstract

**Background:**

In recent years, research on cancer predisposition germline variants has emerged as a prominent field. The identity of somatic mutations is based on a reliable mapping of the patient germline variants. In addition, the statistics of germline variants frequencies in healthy individuals and cancer patients is the basis for seeking candidates for cancer predisposition genes. The Cancer Genome Atlas (TCGA) is one of the main sources of such data, providing a diverse collection of molecular data including deep sequencing for more than 30 types of cancer from > 10,000 patients.

**Methods:**

Our hypothesis in this study is that whole exome sequences from blood samples of cancer patients are not expected to show systematic differences among cancer types. To test this hypothesis, we analyzed common and rare germline variants across six cancer types, covering 2241 samples from TCGA. In our analysis we accounted for inherent variables in the data including the different variant calling protocols, sequencing platforms, and ethnicity.

**Results:**

We report on substantial batch effects in germline variants associated with cancer types. We attribute the effect to the specific sequencing centers that produced the data. Specifically, we measured 30% variability in the number of reported germline variants per sample across sequencing centers. The batch effect is further expressed in nucleotide composition and variant frequencies. Importantly, the batch effect causes substantial differences in germline variant distribution patterns across numerous genes, including prominent cancer predisposition genes such as BRCA1, RET, MAX, and KRAS. For most of known cancer predisposition genes, we found a distinct batch-dependent difference in germline variants.

**Conclusion:**

TCGA germline data is exposed to strong batch effects with substantial variabilities among TCGA sequencing centers. We claim that those batch effects are consequential for numerous TCGA pan-cancer studies. In particular, these effects may compromise the reliability and the potency to detect new cancer predisposition genes. Furthermore, interpretation of pan-cancer analyses should be revisited in view of the source of the genomic data after accounting for the reported batch effects.

**Electronic supplementary material:**

The online version of this article (10.1186/s12885-019-5994-5) contains supplementary material, which is available to authorized users.

## Background

Identifying predisposition variants underlying cancer heritability is of utmost importance and a critical milestone for personalized medicine. Strong evidence for variant contribution to cancer development is evident in tens of genes, many of them are rare. However, a few genes are common enough and thus harboring significant effects at a population level. For example, inherited mutations in BRCA1 and BRCA2 carry high risk for breast and ovarian in women [1–3], prostate in men [4] and pancreatic cancer in both gender [5]. The risk and prevalence of specific germline variants in cancer predisposition genes greatly vary across ethnicities and cancer types, as illustrated by the high prevalence of BRCA1 and BRCA2 variants in Ashkenazi Jews [6, 7]. While each cancer type may have its own signature, a substantial overlap in the identity of known predisposition genes has been observed [8, 9]. Studies of families with high recurrence of cancer identified numerous genes carrying germline mutations with high penetrance (e.g., [3, 10]). The increasing number of sequenced exomes has led to the discovery of additional cancer predisposition genes, mostly with rare mutations [11–13].

In recent years, the task of identifying predisposition variants [3] using data-driven and statistically-sound approaches has become feasible, thanks to the availability of thousands of genomic samples with satisfying sequencing depth and quality, from healthy and diseased individuals (e.g., [9, 14]). The premise is that identifying germline cancer predisposition genes will lead to improved clinical diagnosis of hereditary cancers [15]. The Cancer Genome Atlas (TCGA) [16] is the most exhaustive collection of such data. Batch effects in miRNAs-Seq, RNA-Seq and DNA methylation data from TCGA were reported [17]. However, batch effects in genomic data from whole exome sequencing (WES) were mainly attributed to platform-dependent sequencing reactions and sampling conditions [18]. Additionally, it was noted that TCGA exome sequencing data is liable to inaccuracies resulting from sample calling quality [19] and additional technical effects associated with different batches [20]. The latter is evident through monitoring loss of function (LoF) mutations, and specifically short indels that cause frameshifts [20].

In this study, we performed a detailed analysis of germline variants (common and rare) across six cancer types covering thousands of samples. Our assumption is that germline variants identified using WES of blood samples extracted from cancer patients (excluding leukemia, lymphoma and myeloma cancers) are not expected to show systematic differences across cancer types, assuming that biases attributed to variant calling, indel recording, and population structure are eliminated. Consequently, the reliability and consistency of the data in TCGA can be directly assessed in an analysis avoiding or correcting for such known confounders. In this study, we show that the mapped reads are already subjected to substantial batch effects, and demonstrate the impact of such batch effects on critical statistical measures of the data and pan-cancer downstream interpretation.

## Methods

### Data resource

Approval for access BAM files and clinical data of TCGA cases was obtained from the database of Genotypes and Phenotypes (dbGaP) [21]. We selected a total of 2241 blood derived DNA samples with whole exome sequencing data (Additional file 1: Table S1). We limited the analysis to samples sequenced by the HiSeq-2000 Illumina technology. Aligned sequence data for normal samples in BAM file format and the accompanying metadata was downloaded from GDC portal [22].

### Germline variant calling

Variant calling was limited to exome regions only, as provided by UCSC GRCh38 reference genome [23]. We ran four different variant calling pipelines on each BAM file: GATK ‘HaplotypeCaller’ pipeline v3.5 [24], Atlas2 v1.4.3 [25], Freebayes [26] and Platypus v0.8.1 [27]. We filtered the results by their quality score. Samples with four complete VCF files (for each of the four pipelines) were unified; samples with missing or incomplete VCF files were discarded. Additionally, a conservative protocol was used based on consensus merging, limiting the reported variants to those appearing in at least two variant callers. Running this pipeline on a single BAM file took approximately 22 h and produced a ~ 200 MB unified VCF file.

### Comparing within-gene variant distributions

Many of the presented analyses required comparing the distributions of within-gene variant locations between cancer types. Within each gene, we collected all the called variants (from all samples), and partitioned them into six groups according to the cancer types they had originated from. We considered only the per-gene exomic locations of the variants (e.g. coordinates 0 to ~ 8300 for BRCA1). Denote by L_g,t_ = (L_g,t_(1),..,L_g,t_(k_g,t_)) the collection of the gene exomic locations of all k_g,t_ called variants in a given gene g originating from samples of a given cancer type t. For example, if singleton germline variants were called at nucleotide positions 17, 65, and an additional variant was called at two individuals at position 183 of the KRAS transcript in SKCM (Skin Cutaneous Melanoma) samples, then L_KRAS,SKCM_ = (17,65,183,183). Note that the same locations, or even same variants, may appear multiple times in such a collection (e.g. if a variant is called in multiple samples).

In order to compare two cancer types t,s for a given gene g and obtain a *p*-value for the difference in the distributions of variants within that gene between the two cancer types, we applied a two-sided Kolmogorov-Smirnov (KS) test between the two (cumulative) empirical distributions of the collections, denoting the resulting *p*-values as p_g,(t,s)_ = KS(L_g,t_,L_g,s_).

In order to obtain a final summary measure for the possible presence of batch effect within a gene (with respect to the distribution of variants along it), we took the ratio between the KS p-value of an intra sequencing center pair to the KS p-value of an inter sequencing center pair. Specifically, we defined the ratio r_g_ = p_g,min_/p_g,max_ between the minimum of the *p*-values of BI-BI pairs p_g,min_ = min (p_g,(SKCM,STAD)_, p_g,(SKCM,TCHA)_ p_g,(STAD,TCHA)_) to the maximum of the p-values of BI-WUGCS pairs p_g,max_ = max(p_g,(SKCM,BRCA),_ p_g,(SKCM,UCEC),_ p_g,(STAD,BRCA),_ p_g,(STAD,UCEC),_ p_g,(TCHA,BRCA),_ p_g,(THCA,UCEC),_). We declared a gene to be possibly affected by the batch effect if r_g_ > 1. By taking a minimum-to-maximum ratio, we adopted a conservative criterion for the presence of the batch effect, requiring that all *between*-center *p*-values are smaller than all *within*-centers p-values. As reported, only 33% of the analyzed genes resulted a ratio r_g_ < 1, indicating no batch effect.

## Results

### Ethics approval and consent to participate

Ethical approval for this study was obtained from The Committee for Ethics in Research Involving Human Subjects, For the Faculty of Medicine, Dental Medicine and Life Sciences, The Hebrew University, Jerusalem, Israel (approval number - 29072019).

### Germline variants in exome sequences

In order to test the TCGA dataset for potential batch effects, we processed and analyzed a subset of the cancer-type cohorts in TCGA. We focused on six cancer types, each with at least 250 germline samples (total of 2241 samples): BRCA (Breast Invasive Carcinoma), UCEC (Uterine Corpus Endometrial Carcinoma), STAD (Stomach Adenocarcinoma), SKCM (Skin Cutaneous Melanoma), LIHC (Liver Hepatocellular Carcinoma) and THCA (Thyroid Carcinoma) (Additional file 1: Table S1).

We implemented a unified variant calling pipeline for aligned reads (i.e., TCGA germline BAM files) using conventional, well-accepted variant calling methods (see Methods). We restricted the reported analysis to 1522 samples classified as Caucasian (marked “White” by TCGA) to eliminate possible biases due to ancestry. We also restricted our analysis to samples profiled using Massively Parallel Sequencing (MPS) methodology (only HiSeq) to minimize variations due to the technical genomic data production protocols. As short indels account for the majority of batch effects and inconsistencies [20], they were not included in the variant calling, and only Single Nucleotide Variants (SNVs) were considered.

### Batch effects manifestation in the number of called variants

**Fig. 1:**
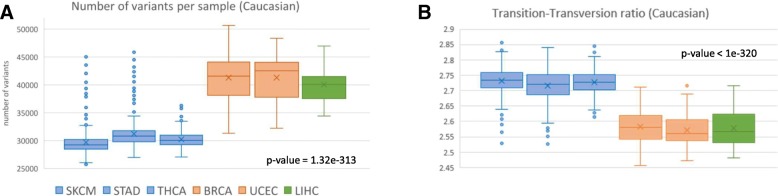
Variability in called variants across TCGA sequencing centers. Batch effect due to sequencing center in 1522 samples associated with Caucasian populations (originated in Europe, Middle East or North Africa) across the six analyzed cancer types. **a** Number of called exome variants per sample. **b** Ratio of transition-transversion (TITv) variants per sample. Colors represent the genomic sequencing centers: BI (blue), WUGSC (orange) and BCM (green)

Our quantitative analysis reveals a significant batch effect in the number of germline variants per sample across different cancer types. The most prominent characteristic shared by cancer types with similar numbers of called variants is the sequencing center contributing to the collection in TCGA (Fig. 1a). The blood samples from patients with skin, stomach and thyroid cancers (SKCM, THCA and STAD) were sequenced at the Broad Institute (BI). Samples from patients with uterus and breast cancers (UCEC and BRCA) were sequenced at the Washington University Genome Sequencing Center (WUGSC) and samples from lung cancer patients (LIHC) were sequenced at the Baylor College of Medicine (BCM) sequencing center.

Numerous aspects of the data analysis are sensitive to the origin of the data, thus reflecting the effect of the different batches. We present several such quantitative measures:

### 20–30% difference in the number of called variants per sample

In Fig. 1 we show the number of called variants per sample, partitioned by the patient’s cancer type. The average number of germline variants greatly varies across sequencing centers. Samples provided by WUGSC and BCM have up to 30% more variants compared to samples provided by BI (one-way ANOVA, *p*-value = 1.32E-313). This observation applies to the other ethnic groups (Additional file 1: Figure S1). Repeating the analysis for each of the four variant callers individually, and applying a conservative consensus-based protocol (see Methods) show the same phenomenon (Additional file 1: Figure S2).

Recently, a report on a catalogue of rare pathogenic germline mutations from TCGA was presented [9]. This report relied on the use of a different variant calling pipeline. The numbers of variants per sample extracted from this report [9] is in a strong agreement with our reported, supporting to notion that the batch effect is insensitive to the underlying variant calling pipeline. Additional file 1: Table S2 provides estimated values for the average number of variants per sample across all 33 cancer types in TCGA [9]. In addition to the three sequencing centers covered in this work, the extracted data also includes a fourth sequencing center, the Sanger center. The overlooked dominating signal of the identity of the sequencing center applies in the data extracted from this report, and generalizes to all 33 cancer types (Additional file 1: Figure S3A). For the six shared cancer types, we report an almost perfect correlation (r = 0.91) between the average number of variants per sample calculated in our analysis to these numbers extracted from Huang, 2018 #242} (Additional file 1: Figure S3B). We conclude that the reported sequencing batch effect dominates the results regardless of the variant calling pipelines used.

### Variations in nucleotide substitution ratios

We find strong evidence for batch effect in the transition-transversion (TiTv) ratios of called variants per sample across sequencing centers (Fig. 1b, one-way ANOVA *p*-value <1E-320). Samples sequenced at BI have ~ 6% higher transition-transversion ratio (average 2.73) compared to samples from the other two sequencing centers (average 2.57). The phenomenon is less prominent where the consensus variant calling protocol was applied (see Methods). Still the batch effect remains statistically significant under the consensus collection protocol (Additional file 1: Figure S2, one-way ANOVA *p*-value 7.49E-51).

### Variant density per gene

**Fig. 2:**
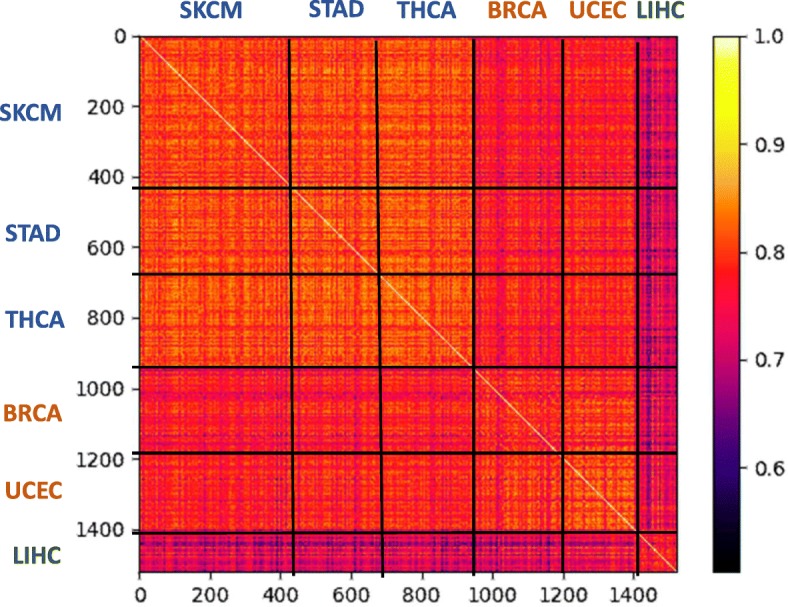
Correlations in variant densities among samples from Caucasian populations. Heatmap of Pearson’s correlations of per-gene variant densities between pairs of samples from Caucasian populations. The 1522 samples are sorted by their cancer types. The correlation values show high similarity among cancer types sequenced by the same canter. The cancer types are as reported in Fig. 1. BRCA (Breast Invasive Carcinoma, 291 samples), UCEC (Uterine Corpus Endometrial Carcinoma, 169 samples), STAD (Stomach Adenocarcinoma, 248 samples), SKCM (Skin Cutaneous Melanoma, 435 samples), LIHC (Liver Hepatocellular Carcinoma, 146 samples) and THCA (Thyroid Carcinoma, 258 samples). Color for the sequencing centers are as in Fig. 1

We addressed the possibility that the observed differences in the number of variants according to the different batches (Fig. 1a) might reflect a naive scaling issue due to different sequencing depths or significance thresholds in variant calling among the sequencing centers. We tested whether the batch effects apply also to the relative variant densities across genes. For each combination of gene and sample, we calculated variant density per nucleotide (i.e., the number of variants divided by the full transcript length). We then calculated Pearson’s correlation for each pair of samples across all transcripts. We show the resulted correlations for the 1522 Caucasian samples (Fig. 2). We find that the batch effect dominates these variant densities, with a strong similarity among samples sequenced at the BI. The lung cancer (LIHC) samples, which were sequenced at BCM, show the largest deviation. We conclude that there are consistent variations among samples from different sequencing centers that are more substantial than naive scaling, leading to enrichment or depletion of called variants in specific genes.

### Variant distribution within cancer predisposition genes

**Fig. 3:**
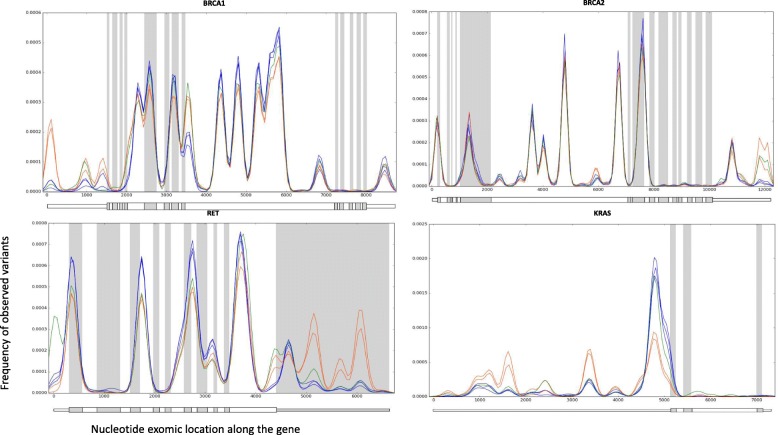
Gene exomic location distributions of germline variants within selected cancer predisposition genes. Empirical probability density functions (PDF) of germline variant coordinates, plotted for four selected genes (BRCA1, BRCA2, RET and KRAS). Each line represents the density function of one of the six cancer types, colored by their corresponding sequencing center: BI (SKCM, STAD and THCA) in blue, WUGSC (BRCA and UCEC) in orange, and BCM (LIHC) in green. The genes BRCA1, RET and KRAS display distinct distributions per sequencing batch, while BRCA2 displays a relatively cohesive distribution. Exons are colored by alternating gray and white backgrounds to enhance the visibility of exon boundaries (introns, for which we have no data, are omitted). The schemas of the transcripts (including the non-coding 5′-UTR and 3′-UTR parts) are shown below each figure. For visibility, the graphs are smoothed by kernel density estimation (KDE), using a window size of 100 nt

We tested whether the batches affect that is attributed to the sequencing centers impact not only the number of variants (Fig. 1) and their distribution *among* genes (Fig. 2), but also their distribution *within* genes. The empirical positional distributions of variant location collections L_g,t_ are displayed in Fig. 3 for all six cancer types within four selected genes. These are representative known cancer predisposition genes: BRCA1, BRCA2, KRAS and RET. We noted marked differences associated with the sequencing centers in the distribution of variants along three of these cancer genes. Interestingly, the strongly reported predisposition gene BRCA2 is mostly indistinguishable for all six cancer types and is thus relatively insensitive to the described batch effect.

**Fig. 4:**
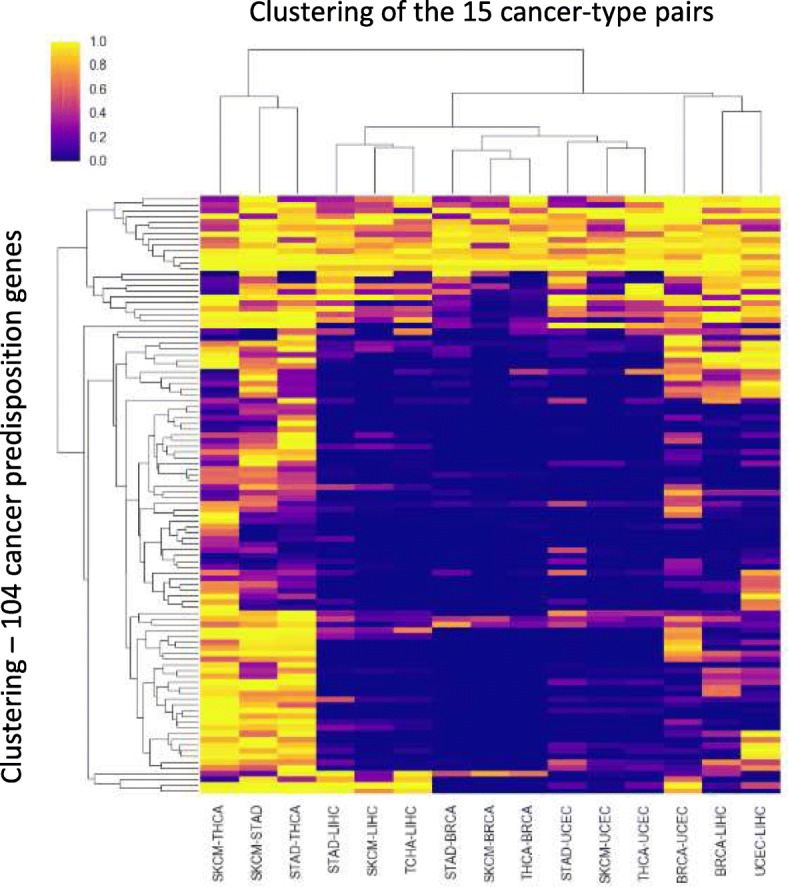
Significant differences in variant location distributions between cancer-type pairs. Bi-clustering of Two-sided Kolmogorov-Smirnov (KS) test results (log-*p*-values) comparing cancer-type pairs across 104 genes annotated by COSMIC as cancer predisposition genes

As illustrated in Fig. 3 for four selected genes, we analyzed the entire collection of 104 known cancer predisposition genes from the COSMIC catalogue [28]. In order to thoroughly quantify the batch effect on the distribution of called variants within those genes (Fig. 4), we used the Kolmogorov-Smirnov (KS) statistical test to compare these distributions between the 15 pairs of the six cancer types for each gene. We clustered the genes and pairs of cancer types based on these statistical results (*p*-values) using Bi-clustering approach. Pairs originating from the same sequencing center were clustered together (e.g., the three leftmost columns corresponding to the three pairs sequenced at BI), highlighting the effect of the sequencing center on variants’ positional distribution.

The susceptibility of genes to the batch effect was determined by the ratio of similarity (using KS *p*-values) *within* and *across* the BI and WUGSC batches (see Methods). Only 35 of the 104 genes were unaffected by the batch effect (*p*-value ratio < 1.0; Additional file 1: Table S3). Variant distribution of genes that are extremely sensitive to the batch effect based on this *p*-value ratio (e.g., MAX, SMACRE1) and other genes that are insensitive to the batch effect (e.g. POLD1) is shown in Additional file 1: Figure S4.

### Batch effects is associated with clinical outcome

We assume that if the identity and distribution of called variants along genes have no impact on pan-cancer downstream clinical interpretation, there will be no difference between genes that are prone to such batch effect and those that are unaffected by it. To test this assumption, we performed an indirect test and followed the survival of patients while focusing on two disjoints gene sets from the 104 genes annotated by COSMIC [28] as germline-associated cancer predisposition genes. Specifically, we sorted the 104 genes by their *p*-value ratio and defined two extreme gene sets: (i) the top 10% (10 genes) that display maximal sensitivity to the batch effect according to the *p*-value ratio: MAX, RET, ERBB4, TSC1, DICER1, BARD1, ERCC5, PRKAR1A, PHOX2B and SMARCE1 and (ii) the bottom 10% (10 genes) showing the minimal sensitivity to such effect: CYLD, POLD1, SMAD4, TSHR, CDC73, NTHL1, SMARCB1, TSC2, FH and SDHD. We performed a survival analysis on cancer patients with somatic mutations from an independent cohort, taken from MSK-IMPACT clinical sequenced samples (MSKCC [29]), which covers 10,129 samples.

**Fig. 5:**
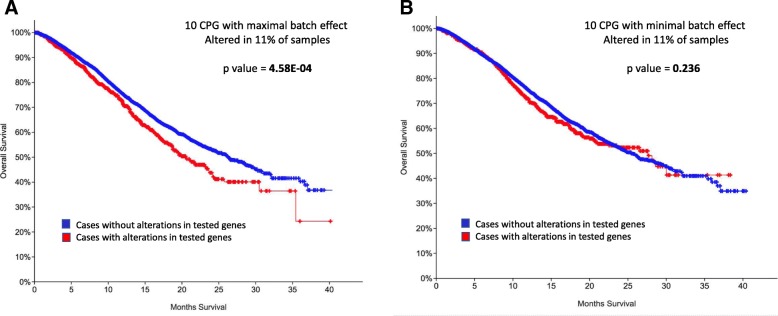
Survival curves for gene sets differing by their sensitivity to batch effect. Kaplan-Meier estimate survival curves tested on 10,129 samples from the MSK-IMPACT clinical sequenced cohort (MSKCC [29]). The analysis applies to genes from a collection of 104 CPGs annotated by COSMIC. **a** Top 10 genes exhibiting maximal sensitivity to the batch effect. **b** Bottom 10 genes exhibiting minimal sensitivity to the batch effect. Additional file 1: Table S3_104 CPG lists the 104 genes along with their batch effect measure

We found a clear difference in the Kaplan-Meier estimated survival curves for the two sets of genes (compare Figs. 5a to b). Specifically, statistically significant reduced survival (Log rank test *p*-value = 4.58e-4, Fig. 5a) is associated with patients carrying mutations in the genes that are maximally sensitive to the batch effect. Such difference is not detected for genes that are resistant to the batch effect (p-value = 0.236, Fig. 5b). In both instances, the fraction of cases with mutations in the gene sets is 11% of all 10,129 samples, showing that the difference in observed effects on survival for the two groups of genes is not due to differences in statistical power. We conclude that the relative sensitivity of genes to batch effect may be carried on to downstream analysis, including clinical outcome and its interpretation, even when one uses independent cohorts for such analysis.

### Batch effects are associated with most of the analyzed genes

**Fig. 6:**
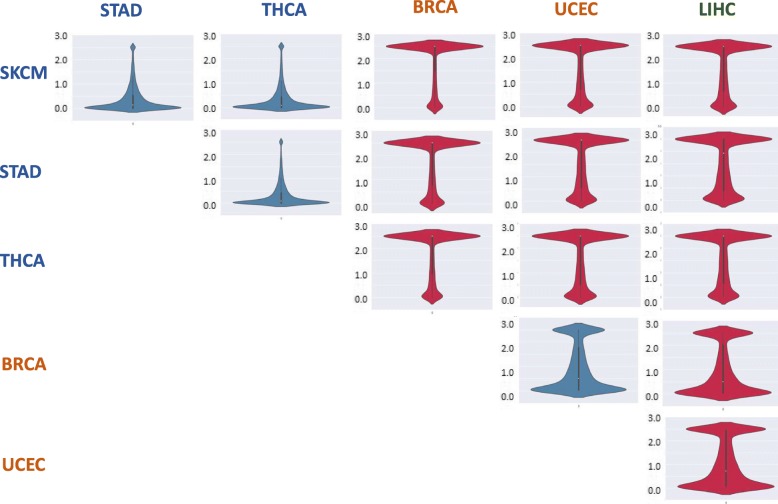
Violin plots based on Kolmogorov-Smirnov test per each group pairing. Two-sided Kolmogorov-Smirnov (KS) tests were carried per gene to test for differences in the distributions of variants between each of the 15 cancer-type pairs (the same variant distributions shown in Fig. 3 for four selected genes). Each panel displays the distribution of resulted p-values across all 18,421 analyzed genes. Red-colored images represent cancer-type pairs originating from different sequencing centers, while blue-colored images represent pairs originating from the same sequencing center. Cancer-type labels are color-coded by sequencing centers, as in all previous figures. The y axis scale is -log10(p-value), where all values above 2.5 were truncated to 2.5 (for visibility)

We expanded the KS paired statistics analysis to include all genes with variants in all six cancer types (overall, 18,421 genes). Only 33% of the genes appear to be insensitive to the batch effect (score < 1; see Methods and Additional file 1: Table S3, all genes). Again, we observe strong similarity between cancer-type pairs sequenced at the same centers, compared to high variability between pairs originating from different sequencing centers. Pairs comparing cancer types from BCM (LIHC) and WUGSC (UCEC and BRCA), as well as the UCEC-BRCA pair show intermediate resemblance (Fig. 6).

## Discussion

We report on multiple layers of batch effects associated with the sequencing centers contributing to TCGA, which are evident upon examination of called germline variants from thousands of samples. These systematic biases raise an urgent need to identify their exact source, be it experimental [30], technical [19, 20] or computational [31]. Understanding the sources of biases is essential for the ongoing effort to mitigate and adjust for such biases from high-throughput collection and data compilation [32, 33].

Somatic mutations in cancer samples exhibit strong characteristics by the cancer type. An entirely opposite trend is expected for germline variants from healthy samples. There, the genomic characteristics signify the ethnic origin of the analyzed samples. By examining samples from the same population (i.e. Caucasians), we expect unified and cohesive genomic signals among samples and across cancer-types. Under such setting, it is easier to isolate the batch effect phenomenon, as we have shown here. However, we anticipate that the batch effect may also infiltrate, to some extent, into somatic mutation analyses, as suggested by our clinical analysis (Fig. 5). However, due to the orders-of-magnitude higher variability in the number of somatic mutations observed among different cancer types, the batch effect is often masked, making it more challenging to identify. Many of the pan-cancer studies performed on TCGA data rely heavily on differences in the total number of somatic mutations among cancer types. Such studies might be skewed due to unaccounted sequencing batch effects. The identity of the genomic centers in which the blood samples were sequenced and the methodology used (i.e., the proportion between samples sequenced by HiSeq technology to data extracted from GeneArray) differ among cancer types and should be accounted for as well.

The reported TCGA batch effect has a broad range of implications. Our results demonstrate similarity among samples originating from the same sequencing center, compared to dissimilarity across samples from different sequencing centers. Our results reaffirm the encompassing nature of the sequencing batch effect that are not restricted to any particular cancer type from TCGA (Additional file 1: Figure S3).

In summary, the observed batch effects influence the number of variants per sample (Fig. 1a), as well as the types of variants (Fig. 1b), the number of variants at a per-gene resolution (Fig. 2), and the distribution of variants within genes (Fig. 3). They also drastically affect the majority of candidate genes annotated as predisposed for cancer (Fig. 4) as well as other genes (Fig. 6, Additional file 1: Table S2).

## Conclusions

The batch effect described in this study is not restricted to the context of cancer, and may affect other human catalogues of WES germline variants. In the context of cancer, the pan-cancer studies are especially prone to batch effect that may lead to false discoveries and misinterpretation. Protocols for determining the identity and prevalence of somatic mutations from patient’s biopsy rely on having an accurate list of its germline variants. Developing methodologies to better control the inherent quantitative imbalances caused by batch effects is urgently and critically needed. Our results suggest that without batch effects correction, pan-cancer analysis cannot guarantee the precision required for personalized medicine. While it is important to apply proper filters to avoid false-positives [34, 35], some of the current filters designed to remove batch effects from whole genome sequencing seem to impede the ability to detect true associations, and find new disease-associated variants [22, 33]. In conclusion, the reported biases underlie the severe discrepancies in germline variants detection and analysis. Additionally, data from the different genomic centers may tamper with detection of somatic mutations, and therefore must be taken into consideration in any data driven pan-cancer analysis and interpretation.

## Additional file

Additional file 1: **Figure S1.** Number of variants in exomes per sample across ethnic groups and cancer types. **Figure S2.** Variability in called variants across TCGA sequencing centers for variants that are in consensus (at least two different variant calling tools.). **Figure S3**. Average number of variants per sample based on an alternative variant calling pipeline (for all 33 cancer types). **Figure S4**. Germline variant distribution plots for representative genes by cancer types. **Table S1.** Number of variants in exomes per sample across ethnic groups and cancer types. **Table S2.** Cancer-type statistics derived from TCGA. **Table S3.** Kolmogorov-Smirnov *P*-value per gene across all pairs of the 6 analyzed cancer types. A measure of the batch distinctive variant distribution pattern is shown for the 104 CPG annotated by COSMIC (named “104 CPG”) and the entire genes (named “all genes”). The table lists all genes with at least a single variant among the compared groups. (ZIP 5414 kb)

## Data Availability

The data that support the findings of this study are available from TCGA (DBGap) but restrictions apply to the availability of these data, which were used under license for the current study, and so are not publicly available. Data are however available from the authors upon reasonable request and with permission of TCGA (DBGap).

## Abbreviations

BCM: Baylor college of medicine
BI: Broad Institute
GSC: Genome sequencing center
KS: Kolmogorov-Smirnov
LoF: Loss of function
MPS: Massively parallel sequencing
SNV: Single nucleotide variant
TCGA: The cancer genome atlas
TiTv: Transition-transversion
WES: Whole exome sequencing
WUGSC: Washington university genome sequencing center
