# Supplementary material for: Substantial batch effects in TCGA exome sequences undermine pan-cancer analysis of germline variants
Source: BMC Cancer. 2019 Aug 7;19:783. doi: 10.1186/s12885-019-5994-5 (PMC6686424; doi:10.1186/s12885-019-5994-5)
Supplement: Supplementary file 1 — Figure S1. Number of variants in exomes per sample across ethnic groups and cancer types. Figure S2. Variability in called variants across TCGA sequencing centers for variants that are in consensus (at least two different variant calling tools.). Figure S3. Average number of variants per sample based on an alternative variant calling pipeline (for all 33 cancer types). Figure S4. Germline variant distribution plots for representative genes by cancer types. Table S1. Number of variants in exomes per sample across ethnic groups and cancer types. Table S2. Cancer-type statistics derived from TCGA. Table S3. Kolmogorov-Smirnov P-value per gene across all pairs of the 6 analyzed cancer types. A measure of the batch distinctive variant distribution pattern is shown for the 104 CPG annotated by COSMIC (named “104 CPG”) and the entire genes (named “all genes”). The table lists all genes with at least a single variant among the compared groups. (ZIP 5414 kb) [file 12885_2019_5994_MOESM1_ESM.zip › TCGA-Suppl Fig S1-S4+ Suppl Tables S1-S2-REVISED-26092018R2.docx]

**Supplementary Information**

**Substantial Batch Effects in TCGA Exome Sequences Undermine Pan-Cancer Analysis of Germline Variants**

Roni Rasnic^1*^, Nadav Brandes^1^, Or Zuk^2^ and Michal Linial^3^

^1^The Rachel and Selim Benin School of Computer Science and Engineering, ^2^Department of Statistics, ^3^Department of Biological Chemistry, Institute of Life Sciences, The Hebrew University of Jerusalem, Jerusalem, Israel

**Figure legends**

**Figure S1.** Number of exome variants per sample across ethnic groups and cancer types (data source: Supplementary Table S1)


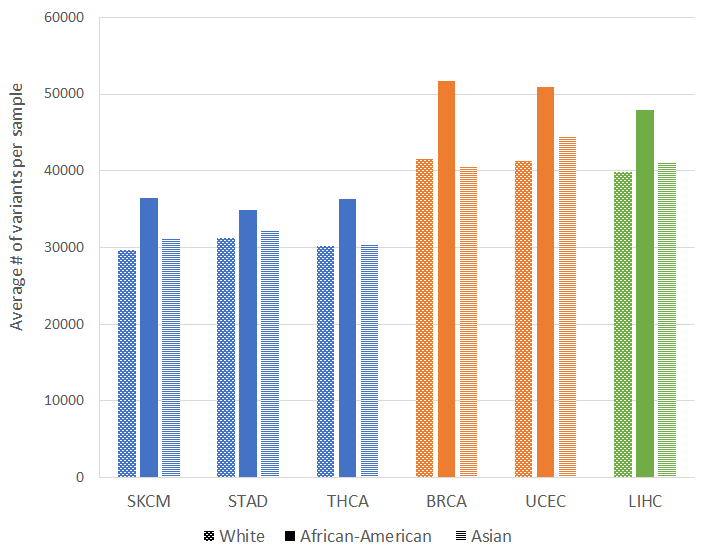


**Figure S2.** Variability in called variants across TCGA sequencing centers in consensus variants.

Batch effect due to sequencing center in 1,522 samples associated with Caucasian populations (originated in Europe, Middle East or North Africa) across the six analyzed cancer types in consensus variants. (**A)** Number of called exome variants per sample. (**B)** Ratio of transition-transversion (TITv) variants per sample. Colors represent the genomic sequencing centers: BI (blue), WUGSC (orange) and BCM (green).

**A**

p-value = 4.48e-318

**
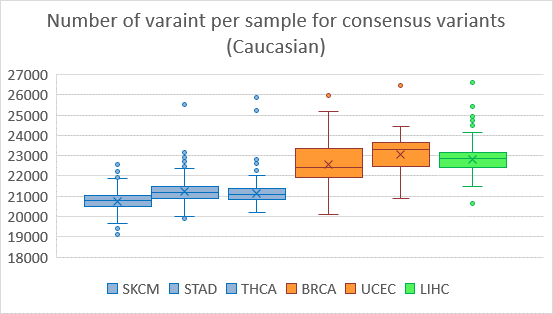
**

p-value = 4.48e-318

**B**

**
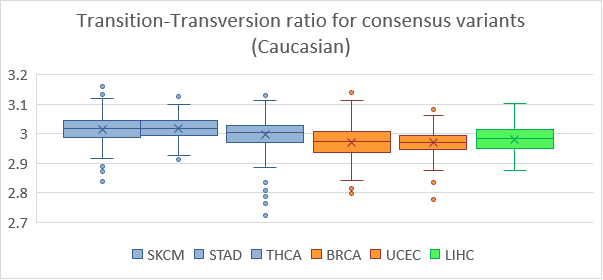
**

p-value = 7.49e-51

**Figure S3.** Average number of variants per sample based on an alternative filtering and variant calling pipeline (for all 33 cancer types)

A


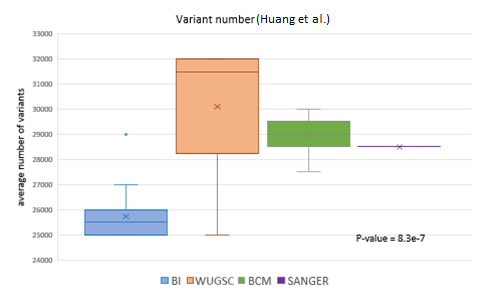


B


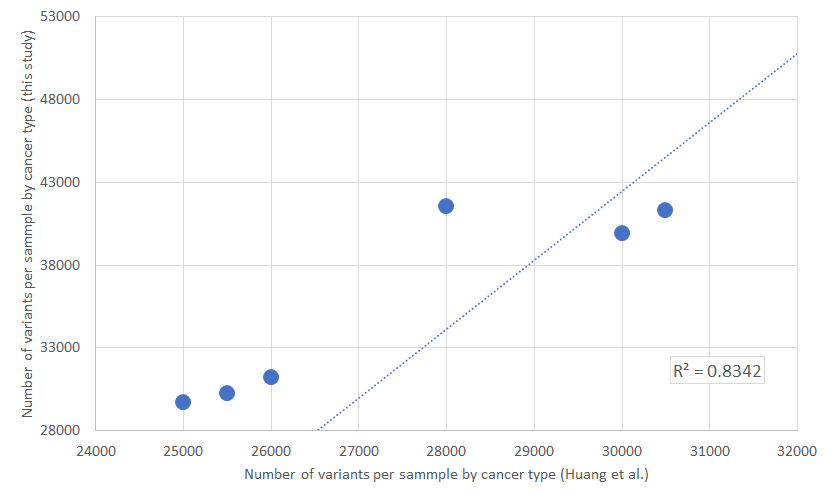


**(A)** Box plots show an average number of variants per sample across all 33 cancer types partitioned by the identity of the four sequencing centers. The data was extracted from another study (Huang et al. [1]; Supplementary Table S2). Boxplots are color-coded by the four sequencing centers that provided the analyzed data: Broad Institute (BI, blue), Washington University Genome Sequencing Center (WUGSC, orange) Baylor College of Medicine (BCM, green) and the Sanger Institute (Sanger, purple). A total of 16 cancer types were sequenced by BI, 9 by WUGSC, 7 by BCM, and 1 cancer type by the Sanger Institute. **(B)** Comparing the values of the 6 cancer types shared by the analysis in this study (y-axis, Supplementary Table S1) to the values in [1] (x-axis).

**References**

1. Huang KL, Mashl RJ, Wu Y, Ritter DI, Wang J, Oh C, Paczkowska M, Reynolds S, Wyczalkowski MA, Oak N *et al*: **Pathogenic Germline Variants in 10,389 Adult Cancers**. *Cell* 2018, **173**(2):355-370 e314.

**Figure S4**. Gene exomic location distributions of germline variants within selected cancer.

Empirical PDF of germline variant coordinates for four representative genes (see Figure 3 in main text). The genes shown are **(A)** MAX, **(B)** SMARCE1, **(C)** BARD1, and **(D)** POLD1. Each colored line represents the distribution for one of the 6 groups. SKCM, STAD, THCA are colored blue; BRCA, UCEC are in orange; LIHC is in green. The distributions per sequencing center are indicated by the score of the KS paired statistics (see Methods). The sensitivity rank is indicated by the percentage (lower percentage indicating higher sensitivity to the batch effect). KS p-values comparing variant distribution among all pairs per gene can be found in Supplementary Table S3 (all genes).


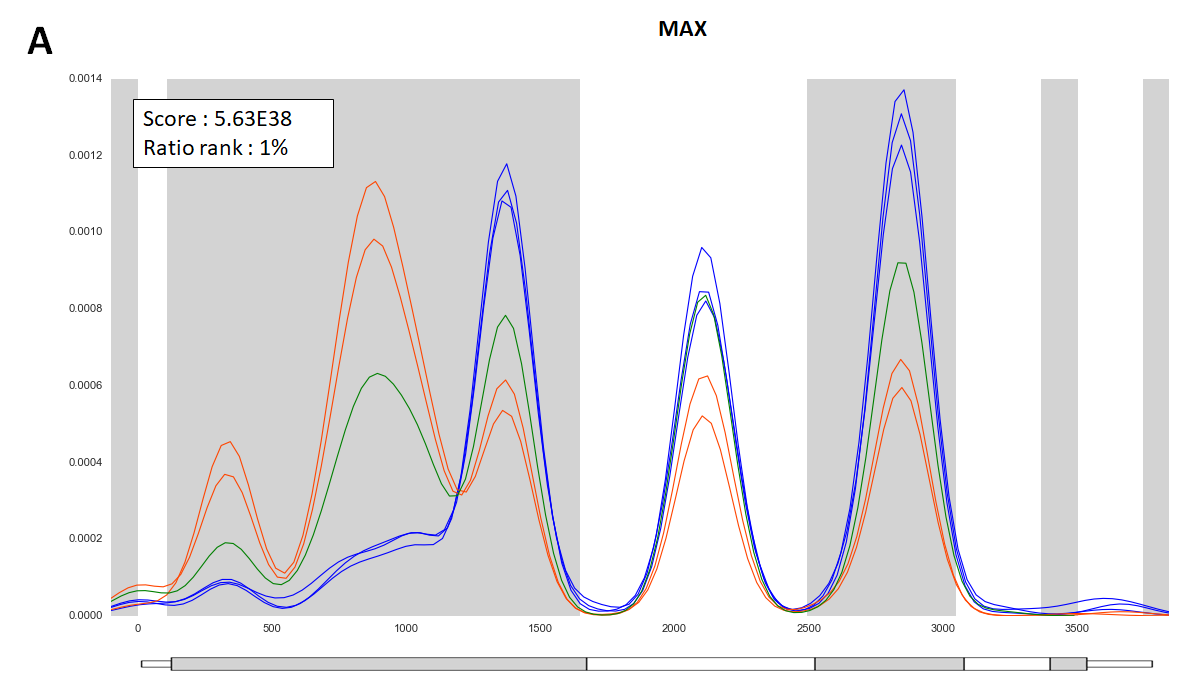


**
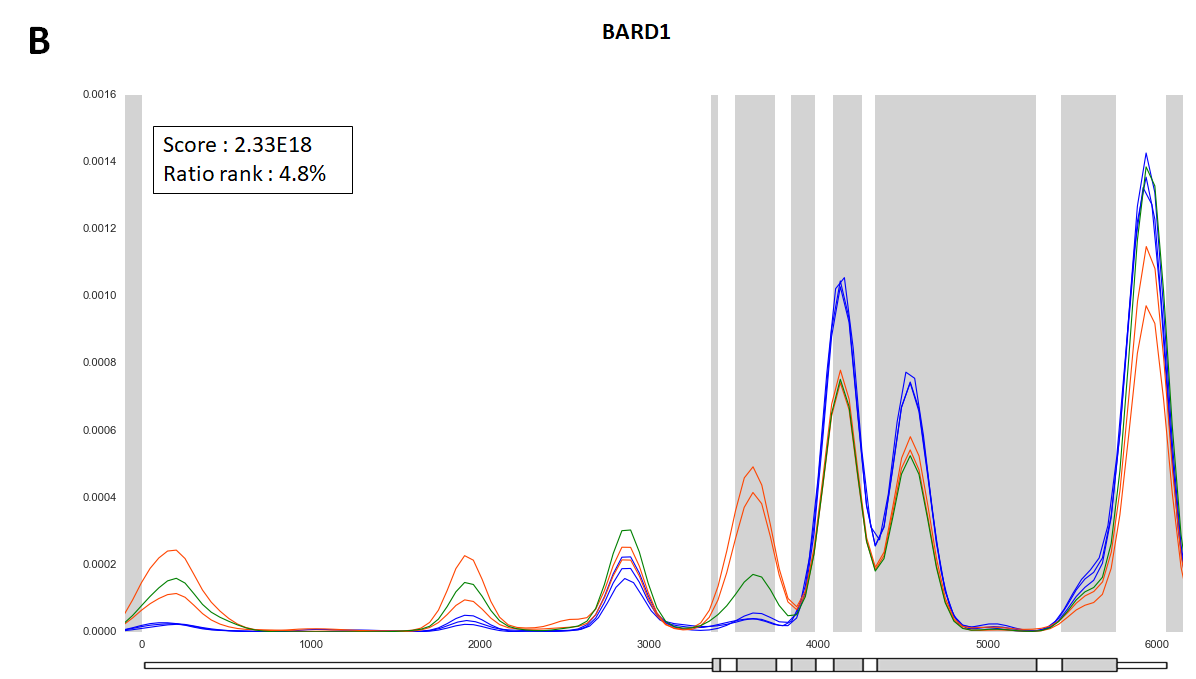
**

**
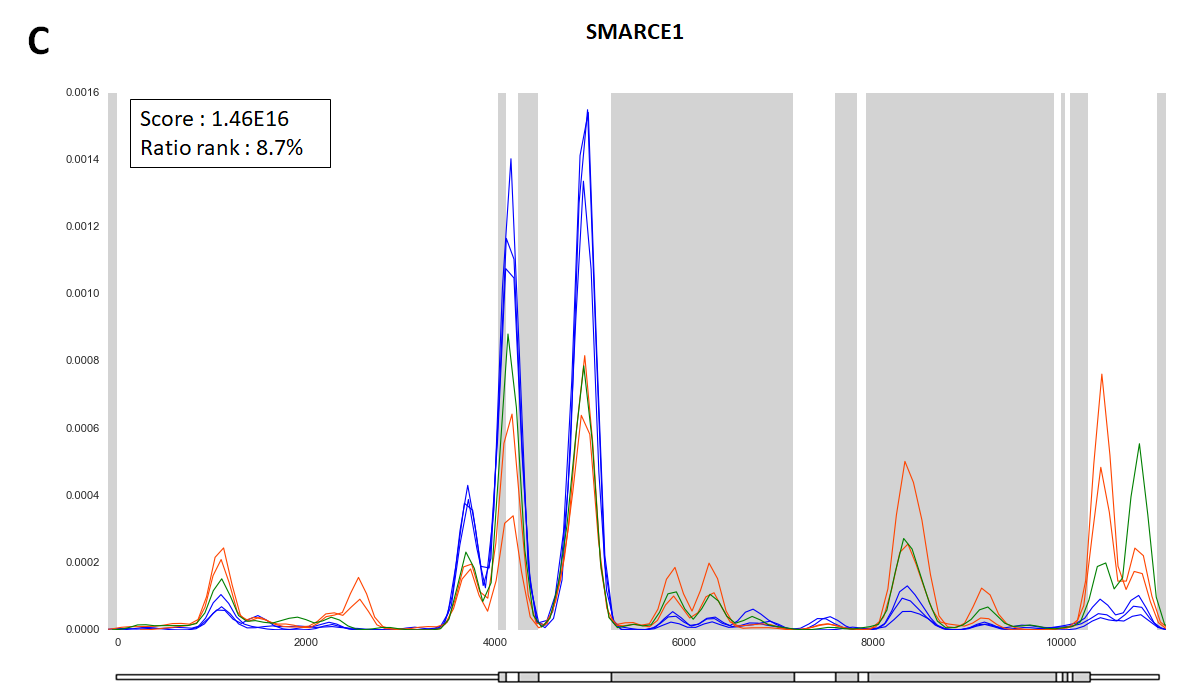
**

**
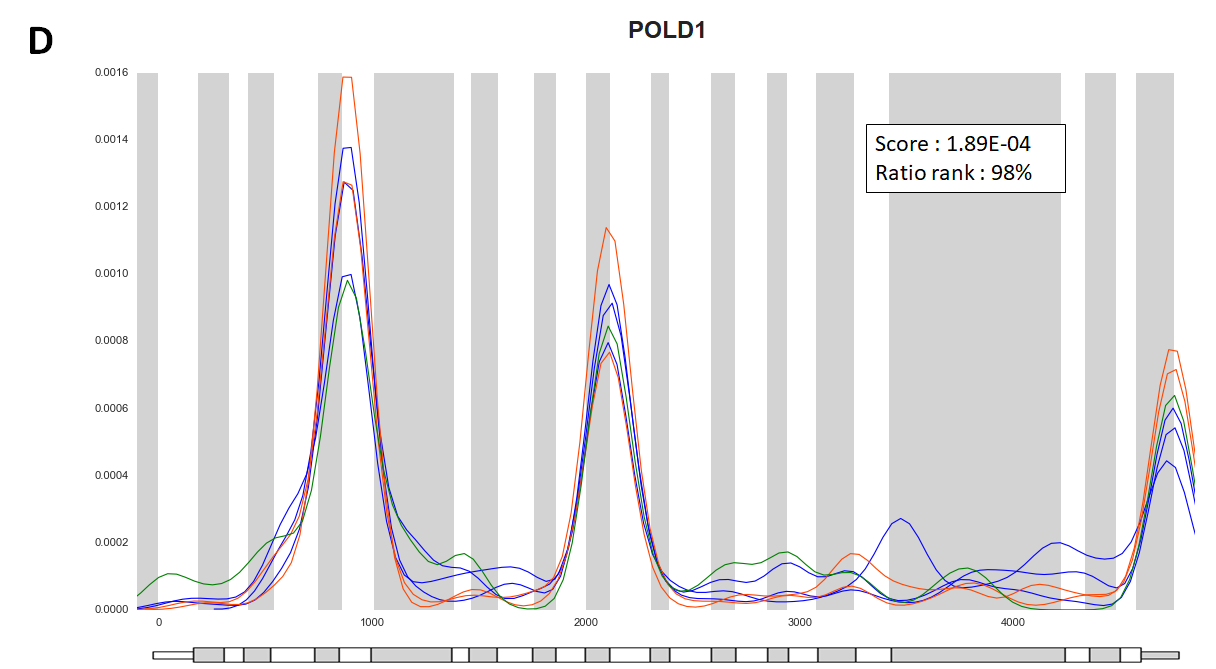
**

**Tables**

**Table S1.** Number of variants in exomes per sample across ethnic groups and cancer types

| **Cancer Type** | **White**  **(Ave)** | **White**  **(std)** | **African-**  **American**  **(Ave)** | **African-**  **American**  **(std)** | **Asian**  **(Ave)** | **Asian**  **(std)** |
| --- | --- | --- | --- | --- | --- | --- |
| **BRCA** | 41561.7 | 4770.1 | 51717.9 | 3304.6 | 40446.3 | 3825.4 |
| **LIHC** | 39884.5 | 3414 | 47976.3 | 5913.1 | 40996.4 | 2593 |
| **SKCM** | 29679.7 | 2382.5 | 36425 | - | 31134.2 | 4087.7 |
| **STAD** | 31228.9 | 3154.9 | 34824.2 | 1142.3 | 32259 | 3540.4 |
| **THCA** | 30251 | 1417.9 | 36294.3 | 1645.9 | 30368.6 | 1310.5 |
| **UCEC** | 41306.7 | 3825.2 | 50976.3 | 4114 | 44563.7 | 6770 |

**Table S2.** Cancer-type statistics derived from TCGA

| **Cancer**  **Type** | **Sequencing Center**^a^ | | **# of Samples** | | **White** | **African** | **Asian** | | **Not provided** | | **Hawaiian / Pacific** | **Indian /Alaska** | **Ave. # variants (Fig. S1E)**^b^ | |
| --- | --- | --- | --- | --- | --- | --- | --- | --- | --- | --- | --- | --- | --- | --- |
| **ACC** | BCM | 92 | | 78 | | 1 | | 2 | | 11 | 0 | 0 | 27500 |  |
| **BLCA** | BI | 412 | | 327 | | 23 | | 44 | | 18 | 0 | 0 | 25000 |  |
| ***BRCA*** | *WUGSC* | *1098* | | *757* | | *183* | | *61* | | *95* | *0* | *1* | *28000* |  |
| **CESC** | WUGSC | 307 | | 211 | | 30 | | 20 | | 36 | 2 | 8 | 31500 |  |
| **CHOL** | WUGSC | 45 | | 38 | | 3 | | 3 | | 1 | 0 | 0 | 31500 |  |
| **COAD** | BCM | 458 | | 213 | | 59 | | 11 | | 174 | 0 | 1 | 29000 |  |
| **DLBC** | BCM | 48 | | 29 | | 18 | | 1 | | 0 | 0 | 0 | 29500 |  |
| **ESCA** | WUGSC | 185 | | 114 | | 5 | | 46 | | 20 | 0 | 0 | 32000 |  |
| **GBM** | BI | 398 | | 341 | | 41 | | 7 | | 9 | 0 | 0 | 25500 |  |
| **HNSC** | BI | 528 | | 452 | | 48 | | 11 | | 15 | 0 | 2 | 25500 |  |
| **KICH** | BCM | 66 | | 58 | | 4 | | 2 | | 2 | 0 | 0 | 29000 |  |
| ***KIRC*** | *BCM* | *535* | | *464* | | *56* | | *8* | | *7* | *0* | *0* | *28500* |  |
| **KIRP** | BI | 291 | | 207 | | 61 | | 6 | | 15 | 0 | 2 | 29000 |  |
| **LAML** | BI | 191 | | 172 | | 15 | | 2 | | 2 | 0 | 0 | 25500 |  |
| **LGG** | BI | 515 | | 475 | | 21 | | 8 | | 10 | 0 | 1 | 25500 |  |
| ***LIHC*** | *BCM* | *377* | | *187* | | *17* | | *161* | | *10* | *0* | *2* | *30000* |  |
| **LUAD** | BI | 519 | | 392 | | 52 | | 8 | | 66 | 0 | 1 | 26000 |  |
| **LUSC** | BI | 504 | | 351 | | 31 | | 9 | | 113 | 0 | 0 | 26000 |  |
| **MESO** | SANGER | 87 | | 85 | | 1 | | 1 | | 0 | 0 | 0 | 28500 |  |
| **OV** | WUGSC | 571 | | 485 | | 34 | | 20 | | 28 | 1 | 3 | 25000 |  |
| **PAAD** | BI | 185 | | 162 | | 7 | | 11 | | 5 | 0 | 0 | 25000 |  |
| **PCPG** | BI | 179 | | 148 | | 20 | | 6 | | 4 | 0 | 1 | 25000 |  |
| **PRAD** | BI | 498 | | 147 | | 7 | | 2 | | 342 | 0 | 0 | 25000 |  |
| **READ** | WUGSC | 170 | | 82 | | 6 | | 1 | | 81 | 0 | 0 | 28500 |  |
| **SARC** | WUGSC | 261 | | 228 | | 18 | | 6 | | 9 | 0 | 0 | 32000 |  |
| ***SKCM*** | *BI* | *470* | | *447* | | *1* | | *12* | | *10* | *0* | *0* | *25000* |  |
| ***STAD*** | *BI* | *443* | | *278* | | *13* | | *89* | | *62* | *1* | *0* | *26000* |  |
| **TGCT** | BCM | 134 | | 119 | | 6 | | 4 | | 5 | 0 | 0 | 29000 |  |
| ***THCA*** | *BI* | *507* | | *334* | | *27* | | *52* | | *93* | *0* | *1* | *25500* |  |
| **THYM** | WUGSC | 124 | | 103 | | 6 | | 13 | | 2 | 0 | 0 | 32000 |  |
| ***UCEC*** | *WUGSC* | *559* | | *374* | | *108* | | *20* | | *32* | *9* | *4* | *30500* |  |
| **UCS** | BI | 57 | | 44 | | 9 | | 3 | | 1 | 0 | 0 | 27000 |  |
| **UVM** | BI | 80 | | 55 | | 0 | | 0 | | 25 | 0 | 0 | 25000 |  |

**^a^**Sequencing centers providing data for 33 cancer types in the TCGA are: Broad Institute (BI), Washington University Genome Sequencing Center (WUGSC), Baylor College of Medicine (BCM) and Sanger center (Sanger). **^b^**Data for number of variants per sample is provided by [12] in supplementary Figure S1E. Average data was presented at a 500 variants’ resolution. In italic are the 6 cancer types analyzed in this study.

**Table S3 -** Kolmogorov-Smirnov P-value per gene across all pairs of 6 cancer types

A measure of the batch distinctive variant distribution pattern is shown for the 104 CPG annotated by COSMIC (named “104 CPG”) and the entire genes (named “all genes”). The table lists all genes with at least a single variant among the compared groups.
